# Sofosbuvir and Velpatasvir Regimen Outcome for Chronic Hepatitis C Patients With End-Stage Renal Disease Undergoing Hemodialysis

**DOI:** 10.7759/cureus.45680

**Published:** 2023-09-21

**Authors:** Salman Shahid, Shoaib Asghar, Tayyab Mahmood, Mishal Fatima, Ali Rasheed, Sohaib Asghar

**Affiliations:** 1 Internal Medicine, Bedfordshire Hospitals NHS (National Health Services) Foundation Trust, Bedford, GBR; 2 Internal Medicine, Sheikh Zayed Medical College/Hospital, Rahim Yar Khan, PAK; 3 Geriatric Medicine, King's College Hospital, London, GBR; 4 Colorectal Surgery, King's College Hospital, London, GBR; 5 Cardiology, Morriston Hospital, Swansea Bay University Health Board, Swansea, GBR

**Keywords:** direct-acting antivirals (daas), chronic hepatitis c (chc), hepatitis c (hcv) infection, cirrhosis of the liver, end stage renal disease (esrd), chronic kidney disease (ckd), velpatasvir (vel), sofosbuvir (sof), hepatitis c management, haemodialysis (hd)

## Abstract

Background

Patients on hemodialysis (HD) are most likely to contract hepatitis C (HCV) infection, which is associated with significant morbidity and disease progression. Direct-acting antivirals (DAAs) are safe and tolerable in chronic kidney disease (CKD) with a 90-100% cure rate, and limited data exist regarding their efficacy in end-stage renal disease (ESRD), particularly for HD patients in South Asia.

The study aimed to assess the outcome of a 12-week sofosbuvir (SOF) and velpatasvir (VEL) treatment regimen on ESRD patients with chronic HCV infection undergoing HD in the Pakistani Asian population.

Methodology

This prospective cohort study was conducted between January 2022 and January 2023 at the outpatient nephrology and gastroenterology clinic of Sheikh Zayed Medical College and Hospital, Rahim Yar Khan, Pakistan. This study included a total of 220 ESRD patients fulfilling the inclusion criteria, aged 20-55 years, who had been undergoing weekly HD sessions for at least two years, with acquired HCV infection.

Data on demographic and clinical characteristics were collected through patient interviews. Laboratory and dialysis profiling was executed to assess ESRD and discover the underlying cause by ultrasound abdomen, blood pressure measurement by sphygmomanometer, random blood sugar for diabetes, and taking note of the duration and frequency of dialysis. HCV RNA PCR was done at selected intervals to evaluate the virological response to treatment. Sustained virological response (SVR), liver cirrhosis status, and number of weekly HD sessions were compared at one year of SOF/VEL regimen.

Results

The mean age of patients with ESRD was 41.8 with a standard deviation (SD) of 9.3 years, and HCV diagnosis was 1.3 years with SD of 0.4 years; 52.7% (n=116) were males, 47.3% (n=104) were females, 75% (n=165) were urban dwellers, and 93.6% (n=206) were married. CKD that requires dialysis was caused mainly by hypertension (78, 35%), diabetes mellitus type 2 (52, 24%), bilateral small kidney disease (40, 18%), and others (34, 16%). One hundred and six (48.2%) received dialysis thrice weekly, 83 (37.7%) twice, and 31 (14.1%) once weekly.

The study monitored the rapid virological response (RVR) at four weeks of SOF/VEL regimen in 89.5% of ESRD patients, observed end-of-treatment response (ETR) at 12 weeks in 93.2%, and noted 91.4% SVR response at one year. Only four (1.8%) relapses were observed in the study, which was statistically insignificant. The status of liver cirrhosis showed a 50% improvement, decreasing from 40% to 20%. The frequency of weekly HD sessions decreased from thrice to twice-thrice a week.

Conclusion

The prevalence of contracting HCV is high among CKD and dialysis ESRD patients. All-oral DAA therapy has revolutionized HCV treatment with co-morbidities. Renal functions improved after the SOF/VEL regimen for chronic HCV infection in ESRD patients undergoing HD, with the number of weekly dialysis sessions reduced and SVR reaching 91.4%. Thus, a single-tablet, pan-genotypic DAA regimen of SOF/VEL for 12 weeks is safe, effective, and tolerable regardless of the underlying etiology of ESRD, complications of cirrhosis, HCV genotype, or previous treatment exposure.

The successful treatment of HCV and achieving SVR lowers the risk of ESRD complications, improves extra-hepatic manifestations, and greatly enhances survival. Further studies are warranted after the availability of other DAAs to confirm findings with no limitations.

## Introduction

Chronic hepatitis C virus (HCV) infection is a major public health issue affecting 58 million globally and results in about 400,000 deaths each year, according to the World Health Organization [1]. In developing countries throughout South Asia, chronic kidney disease (CKD) is highly prevalent. Pakistan, for instance, has an estimated CKD prevalence rate of 23% [2].

The range of end-stage renal disease (ESRD) patients requiring hemodialysis to maintain normal renal function is between 60% and 82%, with advancing age and comorbidities being linked to an earlier and greater need for dialysis [3]. As a result of treatment methods like hemodialysis and kidney transplantation, CKD/ESRD patients are at an increased risk of complications, including myocardial infarction, iron deficiency anemia, fistula formation, osteoporosis, and acquired infections like HCV [4].

Oral interferon‐free direct-acting antivirals (DAAs) have become the first-line standard-of-care treatment of HCV that help reduce inflammation in the liver, slow or stop fibrosis progression, and lower the risks of hepatocellular carcinoma [5]. Among these, pan-genotypic DAAs are widely considered for HCV-infected patients to obtain a sustained virologic response (SVR) due to their tolerability, effectiveness, and affordability [6]. An FDA-approved pyrimidine nucleotide analog called sofosbuvir (SOF) inhibits the ribonucleic acid-dependent RNA polymerase produced by non-structural viral protein 5B (NS5B), whereas velpatasvir (VEL) inhibits non-structural viral protein 5A (NS5A) [7].

The fixed-dose combination of SOF 400 mg and VEL 100 mg is one of the most important pan-genotypic DAA, approved for chronic HCV patients aged ≥6 years, with CKD/ESRD or decompensated liver cirrhosis regardless of HCV genotype [8]. SOF metabolite GS-331007 is excreted through the kidneys and accumulates in patients with impaired renal function [9]. However, the association between SOF-based regimens and renal toxicity remains controversial in clinical trials as no safety concerns were identified [10,11].

The current study examined SOF/VEL regimen outcomes for chronic HCV patients with ESRD who were undergoing hemodialysis in the Pakistani Asian population to better document the outcome of these easily available DAAs.

## Materials and methods

Study design

This prospective cohort study was conducted between January 12, 2022, and January 15, 2023, at the outpatient nephrology and gastroenterology clinic of Sheikh Zayed Medical College and Hospital, Rahim Yar Khan, Pakistan, following approval (286/IRB/SZMC/SZH) from the institutional Ethical Research Review Board. Informed consent was taken and all patient biodata was kept confidential.

The enzyme-linked immunosorbent assay (ELISA) test was utilized to screen all ESRD patients undergoing hemodialysis for HCV antibodies. Those who tested positive received further testing with HCV RNA PCR (qualitative) and were subsequently provided an SOF 400 mg/VEL 100 mg once daily treatment regimen free of charge.

The study included ESRD patients who met certain criteria, which included having a glomerular filtration rate (GFR) of less than 30 mL/min/1.73 m^2^, receiving weekly or thrice-weekly hemodialysis, age between 20 and 55 years, and having a positive qualitative HCV RNA PCR result.

Patients diagnosed with decompensated liver disease, terminal or metastatic malignancy, HIV co-infection, HBV co-infection, intravenous drug abuse, other co-infections (such as Mycobacterium tuberculosis, fungal infections, opportunistic infections), and alcoholism were excluded from the study based on clinical and laboratory findings.

Data collection

The inclusion criteria were met by 220 patients who were selected. Demographic and clinical characteristics were collected through patient interviews, such as age, time since HCV diagnosis, gender, residence, marital status, body mass index (BMI), number of weekly hemodialysis sessions, presence or absence of HCV cirrhosis/ascites, and naïve or pegylated interferon treatment exposure.

The baseline labs were conducted at periodic intervals of four, 12, and 52 weeks post-enrollment into the study. Blood samples were collected and sent to the laboratory for conducting a complete blood count hemoglobin (Hb), prothrombin time (PT), HCV RNA PCR qualitative, liver function tests (LFTs) (alanine aminotransferase [ALT], aspartate aminotransferase [AST], gamma-glutamyl transferase [GGT], alkaline phosphatase [ALP], albumin), renal function tests, and electrolytes (serum creatinine, blood urea, sodium, potassium, bicarbonate, phosphate, and calcium).

Dialysis profiling was executed to discover the cause of underlying ESRD, utilizing an ultrasound abdomen, standard sphygmomanometer for blood pressure measurement, random blood sugar sample for diabetes, and taking note of the duration and frequency of dialysis.

SOF inhibits the ribonucleic acid-dependent RNA polymerase produced by NS5B and VEL inhibits NS5A. The treatment regimen of SOF 400 mg and VEL 100 mg once daily for 12 weeks was assessed in this study. Please note that the medicine was given OFF-LABEL to all patients free of cost. At the time of this study, other DAAs, such as primary HCV treatment options glecaprevir/pibrentasvir, ledipasvir and secondary options voxilaprevir, elbasvir/grazoprevir, were still not available for use in Pakistan.

The primary outcome was to achieve viral elimination from the serum. HCV RNA PCR was done at selected intervals to evaluate the response to treatment. After starting SOF/VEL treatment, rapid virological response (RVR) was seen at four weeks, end-of-treatment response (ETR) was seen at 12 weeks, and sustained virological response (SVR) was evaluated at 52 weeks. The secondary outcome was to assess the cirrhosis liver status, achievement of SVR, and eventual course of hemodialysis sessions needed for ESRD after one year.

Data analysis

The nonprobability convenient sampling technique was used. Data analysis from questionnaires proforma was performed through statistical software IBM SPSS Statistics for Windows, Version 21.0 (IBM Corp., Armonk, New York, United States). Frequencies (%) were measured for qualitative/categorical variables at the start and one year of the study. The mean and standard deviation were measured for age, time since HCV diagnosis, and laboratory variables.

## Results

A total of 220 patients were studied between January 12, 2022, and January 25, 2023; all participants had ESRD with acquired HCV infection and were on maintenance hemodialysis. The mean age of the participants was 41.8 with an SD of 9.3 years, and the mean duration of HCV diagnosis was 1.3 years with an SD of 0.4 years. Among these, 52.7% (n=116) were male and 47.3% (n=104) were female. The majority of people, 165 (75%), were urban dwellers and 206 (93.6%) were married. The average BMI for males was 26.45 ± 0.81 kg/m², whereas BMI for females was 28.73 ± 0.44 kg/m². The majority of ESRD patients (106, 48.2%) underwent hemodialysis three times a week, 83 (37.7%) twice a week, and 31 (14.1%) only once a week. Ascites or cirrhosis was found in 89 (40.4%) patients, and 29 (13.2%) patients failed to respond to pegylated PEG interferon alfa treatment (Table 1).

**Table 1:** Demographic and Clinical Characteristics of End-Stage Renal Disease Patients on Hemodialysis With Acquired Hepatitis C Infection (N=220) BMI, body mass index; ESRD, end-stage renal disease; HCV, hepatitis C infection.

Demographic and clinical characteristics	Categories	N=220 (frequency %)
Age, mean±SD (years)		41.8±9.3
Time since HCV diagnosis, mean±SD (years)		1.3±0.4
Gender	Male	116 (52.7)
Gender	Female	104 (47.3)
Residence	Urban	165 (75)
Residence	Rural	55 (25)
Marital status	Married	206 (93.6)
Marital status	Unmarried	14 (6.4)
BMI (kg/m²), mean±SD	Male	26.45±0.81
BMI (kg/m²), mean±SD	Female	28.73±0.44
ESRD on hemodialysis	Once weekly	31 (14.1)
ESRD on hemodialysis	Twice weekly	83 (37.7)
ESRD on hemodialysis	Thrice weekly	106 (48.2)
Chronic HCV infection	Cirrhosis/ascites present	89 (40.4)
Chronic HCV infection	Cirrhosis/ascites absent	131 (59.6)
Treatment status	Naive	191 (86.8)
Treatment status – PEG interferon alfa	Experienced	29 (13.2)

Among all patients included, RNA PCR demonstrated HCV infection and Hb levels were low, with a mean Hb of 8.36 g/dL with SD of 1.43, which is expected in patients with CKD. PT was 11.2 seconds with an SD of 2.36. A majority of patients required three dialysis sessions per week, explaining the higher creatinine, urea, and potassium levels. The mildly deranged LFTs were representative of chronic liver disease and these alterations seen could be attributed to CKD and HCV infection. All laboratory profiling is provided in Table 2.

**Table 2:** Laboratory Profile of End-Stage Renal Disease Patients on Hemodialysis With Acquired Hepatitis C Infection (N=220)

Laboratory profile	N=220 (mean±standard deviation)
Complete blood count – hemoglobin (g/dL)	8.36±1.43
Prothrombin time (PT) (seconds)	11.2±2.36
HCV RNA PCR qualitative – before treatment	220
Creatinine (mg/dL)	8.78±2.39
Urea (mg/dL)	113.65±35.83
Sodium (mEq/L)	137.4±5.14
Potassium (mEq/L)	4.73±0.84
Bicarbonate (mmol/L)	22.56±2.63
Phosphate (mg/dL)	6.97±1.54
Calcium (mg/dL)	7.87±0.68
Alanine aminotransferase (ALT) (IU/L)	48.29±42.32
Aspartate aminotransferase (AST) (IU/L)	46.63±38.85
Gamma-glutamyl transferase (GGT) (IU/L)	52.34±26.14
Alkaline phosphatase (ALP) (IU/L)	538.51±413.36
Albumin (g/dL)	3.11±0.62

The deranged electrolytes and renal and liver function tests made it necessary for undergoing hemodialysis up to three times a week. According to weekly dialysis session durations, 106 (48.2%) received dialysis three times per week, 83 (37.7%) twice a week, and 31 (14.1%) once per week. CKD that requires dialysis is caused by a wide range of factors, such as hypertension (78, 35%), diabetes mellitus type 2 (52, 24%), bilateral small-size kidney disease (BSSKD) (40, 18%), and others (34, 16%), including echogenic kidneys, chronic tubulointerstitial nephritis, pregnancy-induced renal disease, amyloidosis, and multiple myeloma (Table 3).

**Table 3:** Dialysis Profile of CKD Patients on Hemodialysis With Acquired Hepatitis C Infection (N=220) *Echogenic kidneys, chronic tubulointerstitial nephritis, pregnancy-induced renal disease, amyloidosis, and multiple myeloma. CKD, chronic kidney disease.

Dialysis profile	N=220 (frequency %)
Duration of dialysis - once weekly	31 (14.1)
Duration of dialysis - twice weekly	83 (37.7)
Duration of dialysis - thrice weekly	106 (48.2)
Duration of undergoing dialysis (years), mean±SD	2.9±1.38
Cause of CKD/dialysis - diabetes mellitus 1	9 (04)
Cause of CKD/dialysis - diabetes mellitus 2	52 (24)
Cause of CKD/dialysis - hypertension	78 (35)
Cause of CKD/dialysis - bilateral small-size kidney	40 (18)
Cause of CKD/dialysis - kidney stones	7 (03)
Cause of CKD/dialysis - others*	34 (16)

An RVR to SOF/VEL was monitored at four weeks (RVR), a treatment-end response at 12 weeks (ETR), and a sustained response at 52 weeks/one year (SVR). The majority (91.4%) of patients achieved SVRs with only four relapses noted overall in the entire study, which were statistically insignificant. Primary outcomes are presented in Table 4.

**Table 4:** Primary Outcome – Virological Response After Sofosbuvir/Velpatasvir Treatment (N=220)

Virological response (week monitored)	Treatment achievement status N=220 (frequency %)	
	Yes	No
Rapid virological response (04 weeks)	197 (89.5)	23 (10.5)
End treatment response (12 weeks)	205 (93.2)	15 (6.8)
Sustained virological response (52 weeks)	201 (91.4)	19 (8.6)

The CKD/ESRD patients were followed up after one year of SOF/VEL treatment, with cirrhosis liver status, monitoring of SVR, and number of weekly hemodialysis sessions. These secondary outcomes were compared according to the underlying cause of CKD/ESRD in Table 5.

**Table 5:** Secondary Outcome at 1 Year – Sustained Virological Response/Hemodialysis Course of ESRD and Liver Status (N=220) CKD, chronic kidney disease; ESRD, end-stage renal disease; HD, hemodialysis; BSSKD, bilateral small-size kidney disease. *Echogenic kidneys, chronic tubulointerstitial nephritis, pregnancy-induced renal disease, amyloidosis, and multiple myeloma.

CKD/ESRD cause (N=222)	Sustained virological response		HD course of ESRD at 1 year		Liver status at 1 year	
	Yes	No	Weekly HD - From	Weekly HD - To	Cirrhosis at start	Cirrhosis at 1 year
Hypertension	73	5	Thrice	Twice-Thrice	25	10
Diabetes mellitus 1	8	1	Twice	Once-Twice	2	0
Diabetes mellitus 2	48	4	Thrice	Twice-Thrice	22	11
BSSKD	38	2	Thrice	Twice-Thrice	19	10
Kidney stones	7	0	Once-Thrice	Once-Thrice	2	0
Others*	27	7	Once-Thrice	Once-Thrice	19	13
Overall	201 (91.36%)	19 (8.64%)	Thrice	Twice-Thrice	89 (40.45%)	44 (20%)
Treatment status - naive	178	13	Twice-Thrice	Twice-Thrice	62	23
Treatment status – experienced	23	6	Thrice	Twice-Thrice	27	21

At one year, the liver cirrhosis status improved by 50% from 40% to 20%, the overall survival rate was 95%, and 91.4% of the patients achieved sustained virological remission. The number of weekly hemodialysis sessions dropped from thrice to twice-thrice a week.

## Discussion

In this study, ESRD patients on hemodialysis suffering from HCV infection achieved an SVR of 91.4% by taking SOF/VEL for 12 weeks, with 93.2% achieving ETR and 15 patients failing virologically. Four patients relapsed at one year after reaching ETR. SOF/VEL treatment was generally well tolerated and safe. No serious adverse events or treatment-related discontinuations occurred.

Research conducted in Asian populations, especially Pakistan, tends to focus on PEG-interferon's efficacy versus DAAs when it comes to prevalence, risk factors, genotypes, and treatment outcomes [2,3,11,12]. In Pakistan, every third person on hemodialysis had acquired an HCV infection, according to Akhtar S, et al. [12], who found that active HCV infection was 32.33% prevalent among these patients. It is much higher than the study by Khokhar N, et al. [13]; the prevalence of HCV was reported to be 23.7%, and a history of dialysis longer than two years was an important risk factor. Based on data from the Dialysis Outcomes and Practice Patterns Study conducted in 2019, HCV prevalence among high-income participating countries was almost 13.5%, ranging from 2.6% to 22.9% among patients on hemodialysis [11,13,14].

The majority of participants were male, married, and urban dwellers. No correlation between ESRD and HCV acquisition to BMI could be established, as noticed by Khan MU, et al. [14]. The relatively young mean age of 41.8 years in this study, in keeping with KDIGO clinical practice guidelines, can be attributed to early causes of CKD such as diabetes mellitus, hypertension, BSSKD, echogenic kidneys, chronic tubulointerstitial nephritis, and pregnancy-induced renal disease [15].

In this study, most ESRD patients underwent hemodialysis two (37.7%) or three (48.2%) times a week, with a duration of undergoing dialysis at 2.9 ± 1.38 years and 86.8% were treatment-naive. 13.2% of the patients had treatment experienced with PEG-interferon and developed chronic HCV complications like cirrhosis or ascites (40.4%), as reported by Calvaruso and Craxì [16] and Kim and Song [17]. All of these patients had a mean time since HCV diagnosis of one year and three months and reported negative HCV ELISA report at the start of dialysis.

This study found that more than half of all CKD cases were caused by hypertension or diabetes, which is associated with a high risk of CKD developing ESRD that requires hemodialysis, as suggested by Balk EM, et al. [18] and Ekpanyapong and Reddy [19]. Another disease affecting a younger age group is BSSKD, which is also prevalent. Among the other cases were chronic tubulointerstitial nephritis, echogenic kidneys, amyloidosis, and multiple myeloma [3,20].

By taking oral iron supplements or subcutaneous erythropoietin, the patients were already taking preventive measures against CKD-induced anemia. Their Hb levels were fairly well-controlled, with a guideline target range of 9-11 g/dL. The renal function tests and electrolytes were impaired in keeping with chronic complications of CKD/ESRD [21,22]. The LFTs were indicative of chronic HCV with or without cirrhosis/ascites, and the high ALP levels were not due to HCV but CKD [23]. However, the mean ALP was raised significantly by two cases of multiple myeloma, as observed by Butt N, et al. [3].

The SVR achievement was 91.4% by taking the SOF/VEL regimen for 12 weeks, with only four relapses noted overall at one year of this study, which were statistically insignificant. Prior studies by Huang et al. in Taiwan reported an SVR of 93.4% [24], Spain studies by Latif S, et al. observed an SVR of 92% at 12 weeks [25], Heo J, et al. [20] reported a 98% SVR, while Zhang et al. [26] as well as Li C, et al. [27] reported a 100% SVR. The study in China among kidney transplant patients with HCV-positive donors by Chen R, et al. also suggested 100% SVR [28]. Comparatively, SOF/daclatasvir SVR was reported to be 92.6% by Goel A, et al. [29] and SOF/VEL/voxilaprevir SVR was reported to be 100% by Heo J, et al. [20].

Good results have been seen with DAAs for both treatment-experienced and -naïve patients with a reduction in the frequency of hemodialysis sessions per week from thrice to twice-thrice depending on the underlying etiology [3,8,14,16,20]. Of 86.8% treatment-naïve patients, 80.9% achieved an SVR with improvement in liver status or cirrhosis from 28% to 10%, whereas SVR achievement in 13.2% PEG-interferon alfa treatment-experienced patients was 79.3% with minimal liver status improvement at one year from 12.5% to 9.5%. The highest number of relapses was seen in the hypertension, diabetes, and treatment-experienced groups [17,19].

Patients with active HCV and CKD did not experience liver function deterioration despite having the condition for a while, indicating the importance of early treatment for HCV elimination in reducing mortality rates [6,23,27,30]. No major side effects were reported and the patients did not miss any nephrological consults or dialysis sessions while using DAAs during the study period.

The study had several limitations, including patient exclusion criteria, a small sample size, and no use of renal biopsy to confirm the underlying causes. Additionally, immunocompromised status and a DAA treatment duration of 24 weeks were excluded, and the effects of patients' existing drugs on DAAs were not considered. Finally, nutritional status and interactions between drugs were not fully evaluated. Further studies are warranted to confirm the findings with no limitations.

## Conclusions

The prevalence of contracting HCV is high among CKD and dialysis ESRD patients. All-oral DAA therapy has revolutionized HCV treatment with co-morbidities. Renal functions improved after the SOF/VEL regimen for chronic HCV infection in ESRD patients undergoing hemodialysis, with the number of weekly dialysis sessions reduced and SVR reaching 91.4%. Thus, a single-tablet, pan-genotypic DAA regimen of SOF/VEL for 12 weeks is safe, effective, and tolerable regardless of the underlying etiology of ESRD, complications of cirrhosis, HCV genotype, or previous treatment exposure.

The successful treatment of HCV and achieving SVR lowers the risk of ESRD complications, improves extra-hepatic manifestations, and greatly enhances survival. Further studies are warranted after the availability of other DAAs to confirm findings with no limitations.
